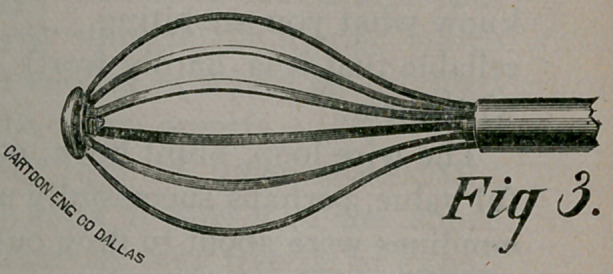# Removal of the Secundines; the Capiat

**Published:** 1891-11

**Authors:** J. S. Poynor

**Affiliations:** Bartlett


### REMOVAL OF THE SECUNDINES.—THE CAPIAT.

J. S. POYNOR, M. D., BARTLETT.

Read before Texas State Medical Association, April, 1891, and published in Transactions.

SOME fifteen years ago the writer was called to see a handsome, pleasant lady, who was dying of septic poisoning caused by retained secundines after a miscarriage or abortion. There was high fever, belly swollen, tense and tender, a stinking discharge from the vagina. It was decided that there was little chance for life, none unless the uterus was cleared of the rotting mass. This was done as well as possible under the circumstances, but the extreme difficulty of even imperfectly clearing the uterus by any means then or since known has made the subject one of abiding interest and study from that day to this. The occasional occurrence of similar cases since has but intensified the interest. A brief consideration of the most approved instrumentalities may not be out of place.

1st. The finger: In cases where the pelvis is shallow, the abdominal walls thin and relaxed, no inflammation, swelling or tenderness, the bimanual plan may be used with some success, but the procedure is coarse, painful and ugly; and when it becomes necessary to ram the hand into the vagina in order to get two fingers into the uterus, the operation can be characterized by no milder term than *brutal*. But the cases in which the finger plan can be prudently and successfully used, probably do not exceed one in a thousand. The idea that any tractile force may be exerted upon the little, slippery placenta or fragments, "the contents hooked out by the finger," as the expression goes, is surely one of the time honored delusions of the profession.

Let anyone make an artificial uterus, as nearly as may be the counterpart of the natural one; place a soft, friable mass and shreds therein, then, with the finger and contents all well oiled, take said uterus in one hand and try to hook out the oiled contents with the oiled finger of the other. If he does not forcibly compress the uterus at the same time he will fully realize the mechanical absurdity of such an attempt. He would note xtract the smallest fragments in a century. Just so in the natural case; if he can not prudently and efficiently squeeze the uterus, which usually he can not, the finger is as useless as would be lubricating the woman's hypogastrium with oleum anseris.

But nearly every practitioner will say: "I have succeeded." Well, yes, we all have, but did we hook it out? Let the above experiment answer. The truth is that the only tractile or expulsive force by the finger or two fingers is on the principle that "two bodies cannot occupy the same space at the same time." As the finger is pushed in, if the uterus has closed down or can be squeezed, the contents will move in the direction of the least resistance, i. e., down by the side of the finger, when the operator will turn his hand so as to bring what he feels in front of his finger, bend it, squeeze as well as he can, withdraw and cherish the delusion that he has "hooked" the thing out. If the brutal procedure of ramming the hand into the vagina and two fingers into the uterus, is resorted to, more force is used, but it differs not in kind from the one finger plan. It is a trifling matter often —two, three or more days after an abortion or miscarriage, when nature has pretty well done the work, when the uterus has contracted and forced the secundines into the cervix and they are extruding from the os—to use the fingers very efficiently; a little pushing from side to side, or effort to pass the finger, will cause the mass to drop into the vagina, as it would soon have done if the woman had got up to the chamber. This is doubtless the reason our fathers authorized, or rather instructed us, to leave these cases to nature rather than do the rough work, and at last probably fail, by any known safe means to afford relief.

But nature often fails, and always acts so tardily that the danger of the fatal sepsis, to say nothing of the numerous other probable evils, is so great while she is acting that the law has gone forth, "clear the uterus." But how? The eminent Professor Goodell, in a lecture on this subject widely published last year, after surveying the field and weapons, repeated the command, but in answer to the *how*, said: "By hook or by crook." Everyone must decide for himself what that means.

The placental forceps is practically worthless. If the jaws are wide enough to grasp with any certainty they are introduced with difficulty, and if the frail, slippery mass is grasped with any firmness it either slips out or is mashed asunder. Or, if the mass has some firmness and projects from the jaws, it slips out as it is drawn through the cervix. Besides, with the forceps you never know what you are biting. In short, placenta forceps are so unreliable that it is hardly worth while to worry and waste time with them.

The wire loop, blunt hook, etc., certainly were of purely fanciful value, perhaps successfully used in a few cases where the secundines were about to drop out anyway—when any plan or anything succeeds.

The curette is an excellent instrument for scraping the walls of cavities. As to removing a little placenta, or fragments, or detached ovum from a relaxed uterus therewith, the problem is about the same as fishing an eel from a tub of water with a weeding hoe. True, if a woman is placed on the Sims position, speculum and all, any amount of blood and stuff may be hoed out if the patience of the patient and operator should endure indefinitely; possibly a little placenta might ultimately be gnawed up and dragged out piecemeal. Or, if the scraping and gouging should provoke contraction of the slightly developed muscular fibres (hardly possible) of the uterus and expulsive efforts of abdominal muscles, a little placenta might be expelled. But, as before said, the practicability of removing a little, slippery, fragile placenta from a relaxed uterus with a curette is about the same as fishing the eel from the tub with the hoe.

This writer has known the operation done by those professing skill, and a day or two afterwards the little thing would drop out, appearing as if a rat had been gnawing it. Other devices have been suggested—suction, etc.,—but those mentioned cover the principal and principles as yet brought forward.

The instrument herewith presented, it is firmly believed, will supply the want as nearly as will ever be possible by mechanical device. Frail, slippery material must be removed in a basket or bag. The basket is presented. Its efficiency is not established by theory alone. Years of experiment and experience by the writer and by professional brethren, a few of whom have had the instrument in actual use, confirm all and more than was expected from it. It has stood the test of actual practice. The name "Capiat" was selected on account of brevity and as having some significance, as those will learn who care to think.

The Capiat designed for intra-uterine use (Figure 1) consists of a tube nine or ten inches long half an inch in diameter, having rings for handles at one end. Through the tube extends a rod, having a ring for a handle. A set of six springs, fitted in distal end, terminating in rounded head, is at the left. Figure 2 shows the same distal end, the rod having been pushed forward, or the tube retracted, thus freeing the springs, which are all lying in the same plane, and which expand into an elliptical or oval form, unless the cavity in which they act should be of less capacity than the spring, in which case the expansion will be limited by the walls of the cavity against which they will rest. Figure 3 represents the same after having rotated the rod from left to right, or tube from right to left, or both simultaneously in these opposite directions, until the tube and rod have made one-third of a complete rotation with reference to each other, when the six springs will be distributed around at euqal spaces, forming a closed basket, which will have enclosed any substance that may have been in the cavity.

The springs may be made of any size, number, strength or capacity that may be desired, but a capacity about that of a common hen's egg will be found most useful, *i. e.*, in a majority of cases. Smaller ones are hardly ever needed; larger ones are occasionally needed.

Three sizes (all to work with same tube and rod), say about hen, turkey and goose egg, would leave little to desire, though there is no reason why sufficient capacity should not be had to take any placenta or ovum as large as they ever pass entire. But after full term pregnancies the muscular layers of the uterus are fully developed, and perform their work well if allowed a little time; if they should not, the hand may be readily passed and the placenta scooped out, though the instrument would be cleaner and less painful. Fragments of the membranes, or placenta, may be readily removed with the Capiat.

Right here it may be remembered that a metallic instrument can be rendered thoroughly aseptic much more readily and surely than the hand. The instrument can be boiled and dipped into powerful antiseptic solutions, while the hand, with its finger nails, cracks and crevices, can not. Neither is the instrument continually getting into dirty places and grasping foul objects, like the hand.

To use the Capiat: It should be thoroughly cleaned, which may be done by boiling or by any of the approved antiseptic solutions, oiled, say with carbolized vaseline, and the various movements made repeatedly to see that it works perfectly. Having the patient in the proper position--it does not much matter which, so that the thighs are drawn out of the way—a finger is passed into the vagina to the os, the os and the cervix being dilated to at least half an inch. No intra-uterine manipulations shonld ever be undertaken with less dilation than this.

The instrument closed, as seen in Figure 1, is then passed along the finger to the os, in and through the cervix up to the fundus of the uterus, which may be determined both by the distance and the resistance to the broad, rounded head of the Capiat.

It is well, after the instrument is in the uterus, to place one hand above the symphisis on the fundus, if practicable, to assist the judgment as to the proper introduction. The head of the instrument having reached the fundus, the rod is held steady while the tube is retracted, thus freeing the springs and allowing them to expand against the uterine walls. If any attachments are apprehended, the tube and rod are held firmly by grasping the rings of both, and gently rotating the entire instrument half around, by which movement the uterine walls will have been swept, except a very small patch directly above the head of the instrument. If desired to sweep this, a little change in the direction of the long axis of the instrument, and a slight turn, will accomplish it. Then distribute the springs form—the basket. After performing the above described movements, any detached or easily detachable substance that may be in the uterus will be enclosed in the basket of springs. The rod may then be held steady and the tube pushed up, thus compressing the springs and sinking them into the soft contained mass, to any entent desired. The dull and easily compressible springs would hurt nothing if withdrawn without compression. In either case the basket and contents are withdrawn at discretion, a finger of one hand being held against the os to make sure that there is no dragging down of the uterus, which should never be done if avoidable. There is not an angle, edge or point about the Capiat that can come in contact with the tissues, hence there can be no laceration.

No very considerable force is ever to be used with the Capiat, either in working it or in working with it. It is abundantly strong for every purpose for which it was intended.

Used rationally, cautiously and gently, it speedily, harmlessly, painlessly and effectually accomplishes its work. A small, round, shallow, dull spoon with a long handle, is the best instrument for severing very firm and extensive attachments, but when attachments are firm and extensive there is little danger of decay or sepsis, and one may very safely wait awhile.

It is hardly worth while to say a word about antiseptic precautions at this late day. Instruments must be clean. By unlocking the French joint at "A," Figure 1, the rod and springs may at once be pushed out of the tube, thus detaching the springs from the rod, and unscrewing the button at the distal end, every piece of the entire instrument is separate, and may be cleaned perfectly. The instrument may be taken apart and put together in a few moments.

The mucous membrane of the uterus (if it may be so called), being firmly attached to the muscular layer, without any intervening submucous or areolar tissue, there is no danger in passing over its surface a dull and easily compressible spring. No danger of lacerating or removing anything that should not come away. Indeed, as experience has proved, this instrument may be used instead of the dull curette, and in the less experienced hands, at least, will do a much better and more expeditous operation than that instrument. If desired, a pair of springs may be sharpened and used effectively in that way, with much more ease, neatness and expedition than the curette proper, though it was not designed for use instead of the curette in its legitimate field.

To recapitulate: The Capiat answers the mechanical problem in theory. Actual use has proven that it does the same in practice.

It will save practitioners, who see proper to use it, from many anxious and weary hours of waiting and temporizing with drugs, tampons and repeated gouging and scraping with uncertain and often dangerous instruments and devices, to say nothing of the tortures and perils which helpless patients will escape, which, after all, is the great matter.

Finally, the writer may be permitted to state that no irreverence is to be inferred from his omitting to refer to eminent authors. He merely exercises the privilege that thirty years of study and average experience ought to confer—that of stating facts observed and conclusions drawn, without circumlocution or apology.

## Figures and Tables

**Fig 1. f1:**
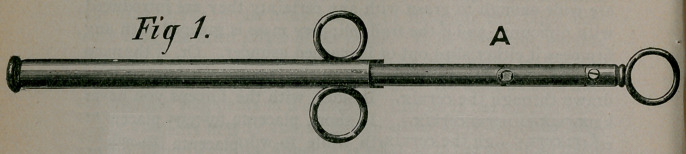


**Fig 2. f2:**
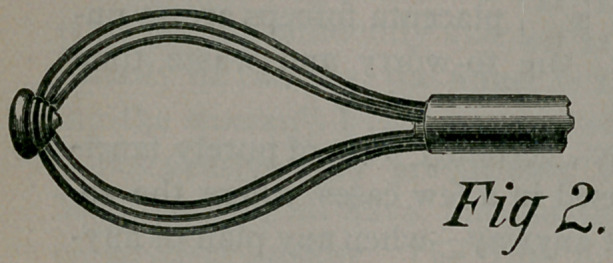


**Fig 3. f3:**